# Supplementary material for: Asynchronous magnetic resonance elastography: Shear wave speed reconstruction using noise correlation of incoherent waves
Source: Magn Reson Med. 2022 Oct 27;89(3):990–1001. doi: 10.1002/mrm.29502 (PMC9792433; doi:10.1002/mrm.29502)
Supplement: Supplementary file 1 — DATA S1 MATLAB code used to generate the numerical simulations of Figure 1 [file MRM-89-990-s002.zip › k-Wave/helpfiles/smooth.html]

smooth :: Functions (k-Wave)


# smooth

Smooth a matrix.

## Syntax

```
mat_sm = smooth(mat)
mat_sm = smooth(mat, restore_max)
mat_sm = smooth(mat, [], window_type)
mat_sm = smooth(mat, restore_max, window_type)
```

## Description

`smooth` filters an input matrix using an n-dimensional frequency domain window created using `getWin`. If no window type is specified, a Blackman window is used.

## Inputs

|  |  |
| --- | --- |
| `mat` | spatial distribution to smooth |

## Optional Inputs

|  |  |
| --- | --- |
| `restore_max` | Boolean controlling whether the maximum value is restored after smoothing (default = `false`) |
| `window_type` | shape of the smoothing window; any valid inputs to `getWin` are supported (default = `'Blackman'`) |

## Outputs

|  |  |
| --- | --- |
| `mat_sm` | smoothed spatial distribution |

## Examples

- 2D FFT Reconstruction For A Line Sensor
- 3D FFT Reconstruction For A Planar Sensor
- 2D Time Reversal For A Line Sensor
- 2D Time Reversal For A Circular Sensor
- 3D Time Reversal For A Planar Sensor
- 3D Time Reversal For A Spherical Sensor
- Image Reconstruction With Directional Sensors
- Image Reconstruction With Bandlimited Sensors
- Image Reconstruction With Compensation For Acoustic Absorption
- Source Smoothing
- Filtering A Delta Function Input Signal
- Modelling Power Law Absorption

## See Also

`fft`, `ifft`, `fft2`, `ifft2`, `fftn`, `ifftn`, `kWaveGrid`
